# Influence of Build Orientation, Chamber Temperature and Infill Pattern on Mechanical Properties of 316L Parts Manufactured by Bound Metal Deposition

**DOI:** 10.3390/ma15031183

**Published:** 2022-02-04

**Authors:** Maitane Gabilondo, Xabier Cearsolo, Mario Arrue, Francisco Castro

**Affiliations:** 1 Department of Additive Manufacturing, IMH Campus, Azkue Auzoa, 1, 20870 Elgoibar, Spain; cearsolo@imh.eus (X.C.); mario@imh.eus (M.A.); 2 Independent Consultant, Gorraiz, 31620 Valle de Egüés, Spain; fcastroceit28@gmail.com

**Keywords:** metal additive manufacturing, bound metal deposition, process parameters, mechanical properties

## Abstract

Bound Metal Deposition (BMD) is an alternative to the most common additive manufacturing (AM) technology for metal parts, Powder Bed Fusion (PBF), since the equipment used is more affordable and there are no risks due to exposure to loose powder and lasers or beams. However, the mechanical properties of parts manufactured by BMD are generally lower than those of PBF, making it necessary to study the process parameters to improve their performance. The aim of this work was to analyse the effect of different process parameters on the mechanical properties of 316L parts manufactured by BMD based on a set of specially designed experiments. The methodology followed in this research was thus based on the manufacturing of a series of samples with variations of the build orientation, infill pattern and chamber temperature followed by subsequent characterization and analysis. The microstructural analysis showed that voids were formed as a consequence of the air gaps generated between rasters during printing. It was observed that the characteristics of these macropores had a significant effect on the mechanical properties. The location, distribution and shape of these macropores depended on the alignment of rasters in each of the conditions, which varied with build orientation and infill pattern. Regarding the build orientation, horizontal parts exhibited lower porosity and considerably higher ultimate tensile strengths (UTS), approximately 160 MPa higher, than vertical samples. With respect to the infill pattern, horizontal parts with a concentric infill pattern showed triangular voids and a total porosity higher than 5%. However, samples with line infill patterns presented elongated macropores and a total porosity lower than 5%, properties that resulted in an improvement in UTS of 20 MPa, approximately. Overall, the results presented here offer a better comprehension of the effect of the BMD process parameters on mechanical properties and serve as a guideline for future work.

## 1. Introduction

In recent years, additive manufacturing technologies have received more attention because they allow complex, light and economical parts to be manufactured with short lead times. These technologies produce three-dimensional parts by successively adding layers of materials such as polymers, ceramics, metals or alloys [1]. Metals in particular are widely used for their superior mechanical properties. Additive manufacturing, when applied to metals, receives the name of metal additive manufacturing (MAM). This technology is becoming an increasingly more important technology in many industrial sectors, such as automotive, aeronautic and medical, because it enables complex parts to be manufactured that cannot be fabricated with other methods [2,3,4,5,6].

The most commercially used MAM processes are those based on PBF, where the powder is spread on a build platform and selectively fused by a high-energy beam. These methods are widely employed mainly due to the high density (usually >99%) and the good mechanical properties of the manufactured parts [1,7]. However, the equipment employed in these technologies is expensive and subjected to strict safety regulations due to risks related to the use of powder and dangerous energy sources such as lasers and beams. Furthermore, the localized melting and rapid solidification characteristics of the PBF method cause high thermal-induced residual stresses, requiring rigid support structures and post processing. In addition, a careful machining step is usually required for support removal [8,9,10].

Extrusion-based MAM methods are presented as an alternative since the equipment used is more affordable and there are no loose powders or lasers during the manufacturing process [5,6]. The manufacturing process of these methods includes several steps, and it usually starts with the preparation of a powder–binder mixture. Then, during the printing stage, which is based on the principle of Fused Deposition Modelling (FDM), the mixture is pushed through a nozzle to deposit material layer by layer and build a 3D object. After printing, it is necessary to perform debinding and sintering to obtain dense metallic parts [1,11].

The formulation of the filament or rod, which is composed of a powder–binder mixture, is an important step. It is necessary to prepare a homogeneous mixture of powder and binder to ensure appropriate flow during extrusion. Powder loadings are usually in the range of 60–80 vol%. In addition to the powder content, the size of the particles is also an important factor. Fine particles with diameters < 20 µm are preferred because they facilitate flow during printing and improve sinterability. However, too small particles should be avoided because they tend to bond and form larger diameter agglomerates. In general, multimodal particle distributions are selected because they allow higher solid loadings, which results in lower sintering distortion and high sintering density. The morphology of the particles is also another important aspect. A spherical shape is preferable since it reduces friction among particles and avoids particle interlocking. The binder system is a multi-component material that is used to shape and bond the powder. There are two main components which correspond to backbone and sacrificial binder. The first one supports and maintains the shape of the part until the final stages of debinding, whereas the second one facilitates flowability during extrusion. The sacrificial binder component is removed in the first stages of debinding, leaving a structure of open pores that allows the diffusion of gaseous products of the backbone in the final stages of debinding. Regarding the composition of the powder, the most commonly used metals are copper, stainless steel (17-4PH, 304L, 316L), Inconel 718, tool steel (M2, SKH10) and titanium (Ti6Al4V) [12].

Extrusion-based MAM methods can be classified into three groups: material extrusion with plungers, filaments and screws [11]. In the case of the first, the material is a bar feedstock made of a mixture of powder and binder. The rod is placed in the guide bush, where the plunger pushes the material into the heater. Then, in the heater, the binder undergoes a melting process until it can be pushed through the nozzle. In the extrusion with filaments, the filaments are rolled, so a feed roller is employed to push the material into the heater. In the same way as in plunger systems, the heater melts the binder, and the material is printed by the nozzle. Finally, material extrusion with screws uses pellets as a raw material. The pellets are transported to the melting zone, where the material is heated, and then to the metering zone, where the molten material is submitted to high pressure before it passes through the nozzle [5,13].

Currently, two companies, Desktop Metal Inc. (DM) and Markforged Inc., offer machines for material extrusion with plungers. Desktop Metal calls their process “Bound Metal Deposition” (BMD), whereas Markforged calls theirs “Atomic Diffusion Additive Manufacturing” (ADAM) [5]. BMD is based on the extrusion of powder-filled thermoplastic media. The raw material is metal rods composed of metal powder held together by wax and polymer binder. The BMD process starts with the upload of a STereoLithography (STL) or computer-aided design (CAD) file in the fabricate software and the subsequent determination of the process parameters. Then, the file is sent to the printing unit, where the rods are heated and extruded onto the build plate, building the part layer by layer. After printing, the binder is removed via solvent and thermal debinding processes. Finally, the parts densify during the sintering cycle. The process uses two extruders; one contains the metal rods that will form the final part, and the other contains ceramic rods that are used as an interface media to separate different parts [14].

BMD is a versatile technology that enables economical metal AM prototypes and low volume production parts to be manufactured in an office-friendly environment. Another advantage is that support removal does not require additional operations because the supports are separated by ceramic interface media, and thus, can be removed by hand [14,15]. However, the parts manufactured with this technology have some limitations in comparison to other methods such as PBF. One of the major concerns is attaining optimum mechanical properties by controlling defects such as high porosity and relatively poor adhesion between layers [16]. These types of defects are usually generated during the printing step, which is based on the principle of FDM technology. Lepoivre et al. [17] determined that the air gaps generated between adjacent circular rasters in FDM resulted in macropores that decreased the mechanical properties. Syrlybayev et al. [13] reported that increasing the temperature during the printing step may improve the adhesion between adjacent layers and also reduce the porosity. Wahab Hashmi et al. [18] and Ligon et al. [19] stated that the process temperature, among other parameters such as raster angle, may influence the part quality, and thus the final properties.

Therefore, an appropriate control of the process parameters is necessary to minimize defects that may lead to inadequate performance in BMD. In addition, there are only a few reports on this technology [15,20], which do not contribute to establishing the relationship between process and properties, and thus a wider use of this technology in the manufacturing of commercial parts. The aim of this work is to study the process parameters that may have a greater effect on the formation of defects in order to establish a relationship between process parameters and mechanical properties in BMD. The presented approach includes the manufacturing of BMD parts with different orientations and printing parameters, including chamber temperature and infill pattern. The subsequent characterization of the manufactured samples consisted of microstructural analysis, porosity measurements and hardness and tensile testing.

## 2. Materials and Methods

This section summarises the material used and experimental procedure followed in this work. Its main purpose is to establish the relationship of build orientation, chamber temperature and infill pattern with mechanical performance.

**Figure 1:**
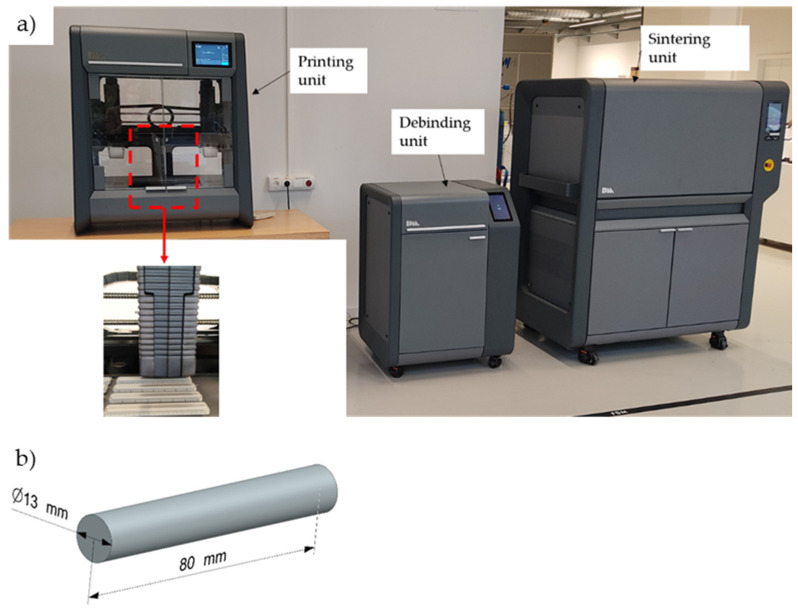
The equipment used in experiments and the cylindrical parts: (**a**) image of the three units of the Studio System^TM^ used for manufacturing of 316L stainless steel cylindrical parts and (**b**) the design (geometry) of the cylindrical parts.

First, cylindrical parts of 316L stainless steel were manufactured using the Studio System^TM^ (Figure 1a, Desktop Metal, Burlington, VT, USA)**.** This system consists of three units which are used sequentially; it starts by printing, followed by solvent debinding and finally, sintering in a high-temperature furnace with controlled atmosphere. The dimensions of the cylinders has diameters of 13 mm and lengths of 80 mm (Figure 1b), based on the user guide provided by DM and ASTM E8 [21].

The CAD design was uploaded to the fabricate software, which is incorporated in the Studio System for part preparation. The standard profile used in this work had default values for sample printing, which can be adjusted according to needs. As the purpose of the research was to study the influence of process parameters that are relevant to the mechanical performance of the parts, cylinders with different printing conditions were prepared. The studied parameters were the build orientation, chamber temperature and infill pattern.

### 2.1. Build Orientation

**Figure 2:**
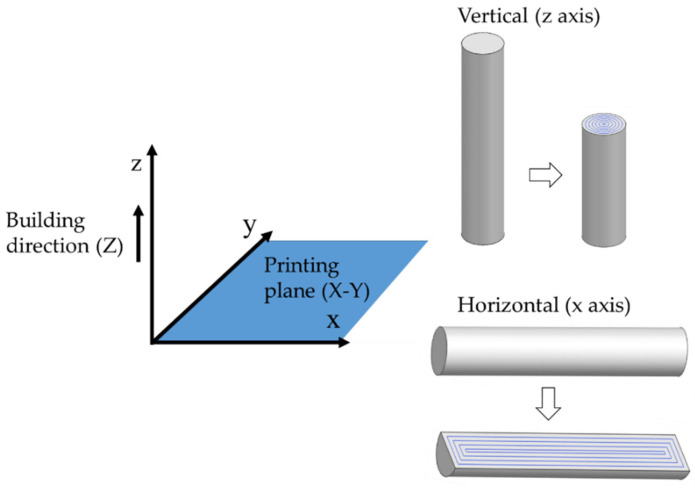
Infill pattern of the samples oriented in X and Z axes.

The cylinders were oriented on the X (horizontal) and Z (vertical) axes to investigate the effect of build orientation on the final properties. The resultant infill pattern for each orientation is presented in Figure 2. It shows that the layers of horizontal parts are composed of concentric rectangles and the layers of vertical samples are circumferences. The sample oriented along the X direction was composed of 101 layers (124 in total, taking into account the support), whereas the part manufactured along the Z direction presented 613 layers (635 in total).

### 2.2. Chamber Temperature

The effect of chamber temperature was studied in both orientations, X and Z, and the values were room temperature and 50 °C, which correspond to the only two options in the fabricate software. It was decided to raise the chamber temperature because it was reported in the literature that increasing the temperature of the printing chamber may improve the part quality, especially adhesion between layers and density [13,18,19].

### 2.3. Infill Pattern

**Figure 3:**
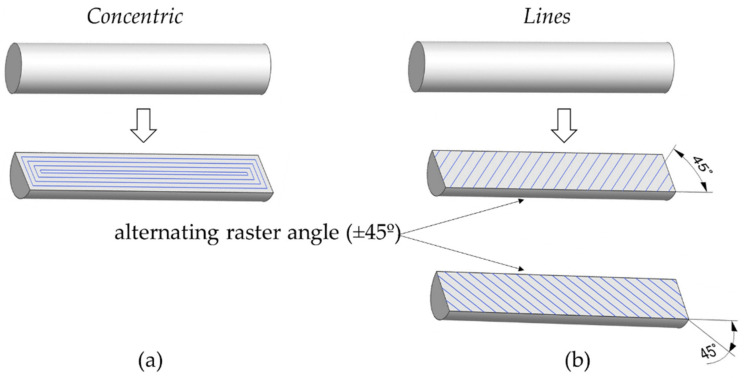
Design of the parts with (**a**) concentric and (**b**) lines infill pattern.

Two different infill patterns were investigated for horizontal parts (Figure 3). The first one was the infill pattern called concentric in the fabricate software and corresponded to the pattern used in Section 2.1 and Section 2.2. The other one was defined as lines and resulted in an infill pattern with alternating raster angles of +45°/−45° between layers.

Table 1 summarizes the conditions used in the different experiments carried out in the Studio System printer.

After printing, the debinding and sintering processes were carried out in the debinding unit and the furnace of Studio System. The fabricate software automatically determines the parameters of these processes to obtain a successful part. The solvent debinding process consists of two stages: immersion of the components in solvent and drying. Both operations were carried out inside the Studio System debinding unit. In the first stage, the parts were immersed in the debind fluid trans-1,2-dichloroethylene, which was heated up to 44 °C. Then, the parts were dried by heating them to 70 °C. Later, the parts were taken to the Studio System furnace, where the parts were thermally debound and sintered in a controlled atmosphere of 3% hydrogen and 97% argon.

### 2.4. Characterization

Six cylinders were manufactured for each of the conditions. First, nine samples were cut from three specimens using Electrical Discharge Machining (EDM, Ona Electroerosion, Durango, Spain). After cutting, they were prepared for density measurement by ultrasonic cleaning and drying at 80 °C to remove any moisture. The density was determined using the Archimedes principle by weighing in air and in distilled water. A Serie Gram FD-140 balance (Gram, l’Hospitalet de Llobregat, Spain) with a specific density measurement device for solid materials was used to conduct density measurements. The balance had a measuring accuracy of 1 mg. The density of the parts was calculated using Equation (1) [22]:

(1) $$\rho_{p}=\frac{m_{\mathrm{air}}}{m_{\mathrm{air}}-m_{\mathrm{fl}}}\cdot \rho_{\mathrm{fl}}$$

where $\rho_{\mathrm{fl}}$ is the density of the fluid, $m_{\mathrm{air}}$ is the mass of the specimen in air and $m_{\mathrm{fl}}$ is the mass of the part in fluid. The actual density and porosity percentage were calculated considering the theoretical density of 7.96 g/cm^3^ of the AISI 316L [23].

**Figure 4:**
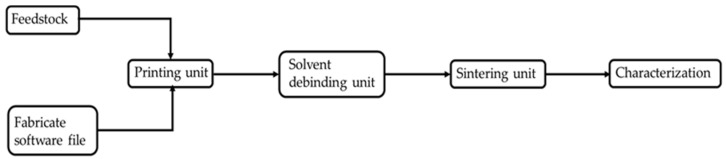
Manufacturing process diagram.

In addition, parts were sectioned, mounted and polished for cross-sectional analysis. The polished sections were etched in a solution of HCl and HNO_3_. An optodigital microscope (Leica Microsystems, l’Hospitalet de Llobregat, Spain) was used for micrographic analysis. Vickers microhardness was measured using a Matsuzawa MHT-1 tester (Matsuzawa, Akita, Japan) under a load of 1000 g. Three samples were analysed for each of the experiments, and five microindentations were made in each of them. Figure 4 shows the manufacturing process diagram.

**Figure 5:**
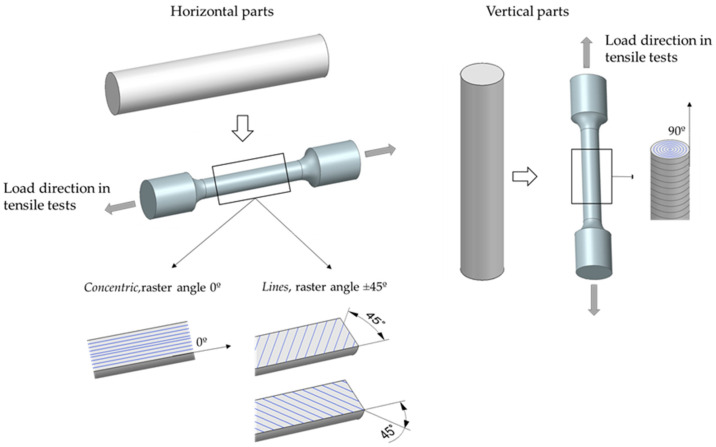
Horizontal and vertical tensile test specimens according to ASTM E8.

Manufactured parts were machined for tensile tests according to ASTM E8. The tensile testing system was a universal servohidraulic Instron 8034H0316 (Instron, Norwood, MA, USA) with a 500 kN load cell. All the tests were conducted in the axial direction, as shown in Figure 5. It can be seen that the alignment of the rasters with respect to the direction of applied load varies with build orientation and infill pattern. In the case of horizontal and concentric parts (experiments 1 and 3), the rasters have an angle of 0°, being parallel to the direction of load. For horizontal specimens with a lines infill pattern (experiment 5), the raster angle varies every other layer from +45° to −45°. Finally, in vertical parts (experiments 2 and 4), the resultant raster angle is 90°, so that the applied load is normal to the layers.

## 3. Results and Discussion

### 3.1. As-Built Parts

**Figure 6:**
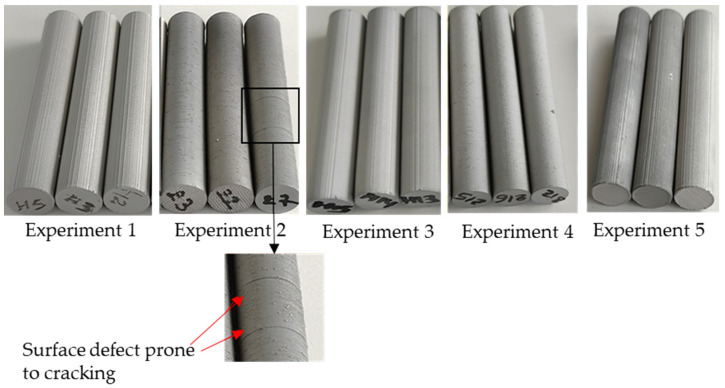
The macroscopic aspects of obtained cylindrical parts from experiments 1–5 and details of surface defect from experiment 2.

Figure 6 shows the general aspect of the parts. All parts showed a high surface roughness, which may be due to the staircase effect that is typically observed in layer-by-layer processes [18,19]. Furthermore, the surface roughness was more evident in the horizontal parts (experiments 1, 3 and 5) than in vertical ones. This may be attributed to the difference in the build angle, as reported in the literature [24,25]. In the vertical parts, all the layers were parallel circumferences, with the angle being 0°. In the horizontal cylinders, however, the dimensions of the rectangles of each layer were different, resulting in a higher angle value and, thus, a higher surface roughness.

On the surface of some vertical parts (experiment 2), defects prone to cracking can also be observed. These defects were the result of the printing process, which was periodic. When several pieces are printed at the same time, the system does not print the full part at once. In this case, the printing sequence proceeds to print a number of layers per piece, thus partially printing each of them, before coming back to the beginning and repeating the process until the pieces are fully constructed. As a consequence, the new layers are printed at each process repetition on previously deposited layers, which had cooled down, thus resulting in potentially poor adhesion. These defects are generated at random as they depend on different factors such as the location of the pieces in the chamber, the number of pieces being printed or the temperature gradients in the printing volume.

**Figure 7:**
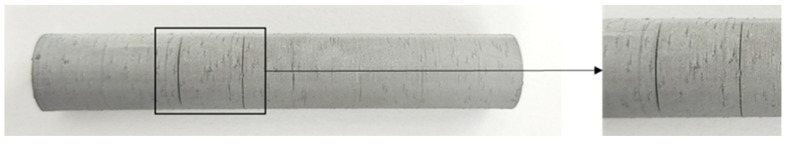
Cracking in the parts manufactured in experiment 2.

As Figure 7 shows, some of these surface defects may result in cracking in the upper surface (surface in contact with gas flow in the furnace) of some parts of experiment 2 after sintering. This phenomenon may have been produced as a result of shrinkage and thermal gradients during sintering. In the vicinity of the surface defects, the tensile stress produced by these effects may also result in cracking. During the thermal treatment, the cylinders are subjected to significant shrinkage as a result of pore removal after debinding. It may be relevant to mention that the raw material used in BMD is a rod consisting of metallic particles embedded in a binder mixture of organic ingredients in a concentration of around 40%vol. In addition, there might be a considerable thermal gradient along the cross section of the cylinders because the upper surface is in contact with the gas flow, while the bottom surface is always in contact with the tray base. As a summary, the shrinkage and thermal gradients result in stresses that may produce cracking in some cylinders.

The fact that this phenomenon was only observed in experiment 2 may be related to the parameters of build orientation and printing temperature in the chamber. Referring to the orientation, the cracks observed in experiment 2 (Figure 7) were produced at the interface of two layers, which indicates that the stresses generated in the longitudinal direction during sintering were larger than the bonding force between layers. Although there may be stresses along the whole cylinder, they might be more important in the longest direction, the longitudinal direction, which was perpendicular to the direction of crack formation. In the case of horizontal parts (experiment 1), stresses along the longitudinal direction may also be high, but they did not lead to cracking because the deposited lines were aligned with the direction of highest stress.

Regarding the printing temperature, the parts manufactured in experiment 4, which were printed at a higher temperature than experiment 2, did not show cracks. Previous reports on FDM show that increasing the temperature of the previous layer can result in better adhesion between layers because more polymer diffuses across the boundary [26]. Therefore, the higher printing temperature of the chamber in experiment 4 may result in better bonding between layers.

### 3.2. Microstructural Analysis

**Figure 8:**
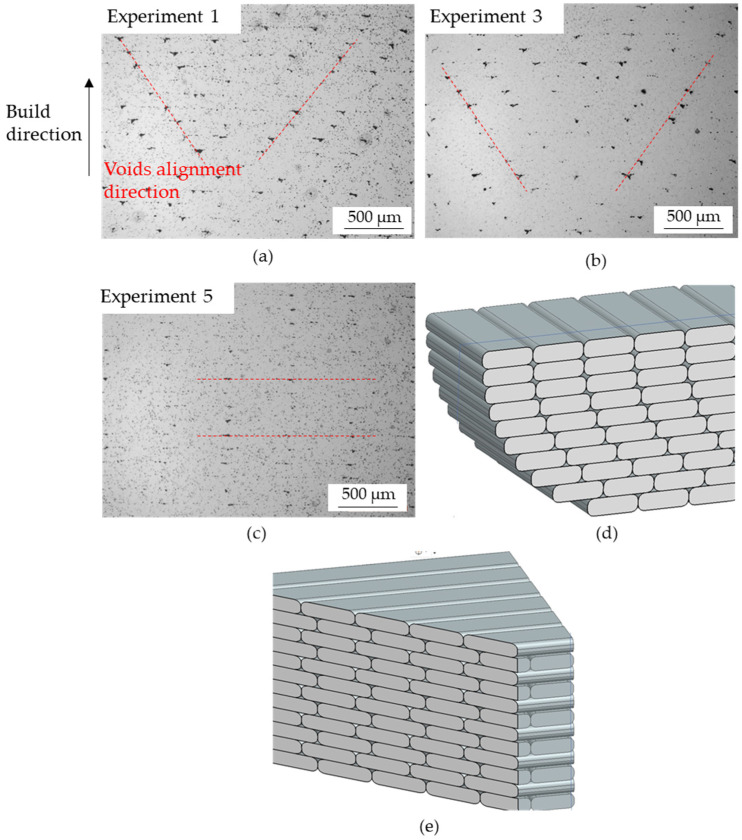
Opto-digital cross-sectional micrographs of (**a**) experiment 1, (**b**) experiment 3, (**c**) experiment 5 and raster orientation in (**d**) experiments 1 and 3 and (**e**) experiment 5.

The cross-section of the horizontal parts manufactured in experiments 1, 3 and 5 was analysed using optical microscopy (Figure 8). It was observed that the cross-section of the samples of experiments 1 (room temperature) and 3 (50 °C) showed triangular macroporosity (voids) with sharp edges at interstitial sites, typical from FDM [27,28], with average sizes, standard deviations and maximum values of 57 ± 8 and 70 µm, respectively. There were no significant differences between both experiments in the size and distribution of these voids, which may indicate that the temperature jump was not enough to observe an effect in these types of defects.

In contrast, in experiment 5, the macropores mainly had an elongated shape instead of triangular. This difference in the geometry of the voids may be attributed to the infill pattern because they were formed as a consequence of the air gaps generated between the rasters during printing. As observed in Figure 3, the infill pattern for experiments 1 and 3 was concentric. In this infill pattern, the layers were slightly displaced with respect to previous ones in order to generate the circular form in the cross-section, which resulted in the formation of triangular voids in the air gaps between the rasters of adjacent layers (Figure 8d). Figure 8a,b show that the displacement between adjacent layers favoured an inclined alignment of the voids. However, in experiment 5, the infill pattern was lines (Figure 3) with an alternating raster angle of ±45°, which resulted in an angle of 90° between adjacent layers (Figure 8e). As the adjacent layers were perpendicular to each other, the resultant geometry of the voids was elongated, and they were horizontally aligned, as seen in Figure 8c. Therefore, the difference in the orientation between adjacent layers produced by the infill pattern was translated into a difference in void alignment.

During the design preparation step, a layer height value of 150 µm was specified in the fabricate software for the standard mode. The layer height measured experimentally in the as-sintered samples of Figure 8 was 120 µm, which agrees with the 15–20% volume contraction expected in the BMD process.

**Figure 9:**
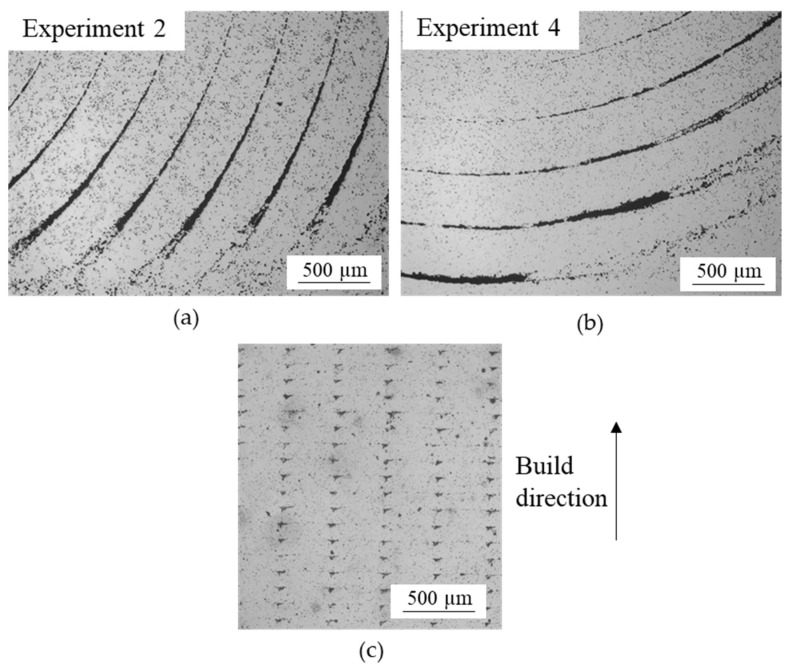
Opto-digital cross-sectional micrographs of (**a**) experiment 2, (**b**) experiment 4 and (**c**) longitudinal section of vertical parts.

The cross-sections of vertical parts (experiments 2 and 4) are shown in Figure 9a,b. Macropores can be observed in both cases and there are no significant differences regarding their size and distribution, even though the temperature of the chamber was different. Therefore, as in horizontal samples, chamber temperature does not appear to have a significant effect on voids.

Figure 9 shows that the voids in the cross-section were aligned in concentric circles, which revealed that air gaps were generated between adjacent circumferences of the concentric infill pattern illustrated in Figure 2. The distance between the circumferences of voids was 360 µm approximately, which agreed with the line width of fabricate (480 µm) and the expected shrinkage in BMD (15–20%). The longitudinal section of the vertical samples is also shown in order to analyse the shape of the voids more closely (Figure 9c).

The voids generated by the air gaps produced during printing and observed in the parts manufactured in all experiments were not completely removed during sintering due to their large size. Their presence is undesirable because they negatively influence the mechanical properties, and consequently, the performance of the parts.

**Figure 10:**
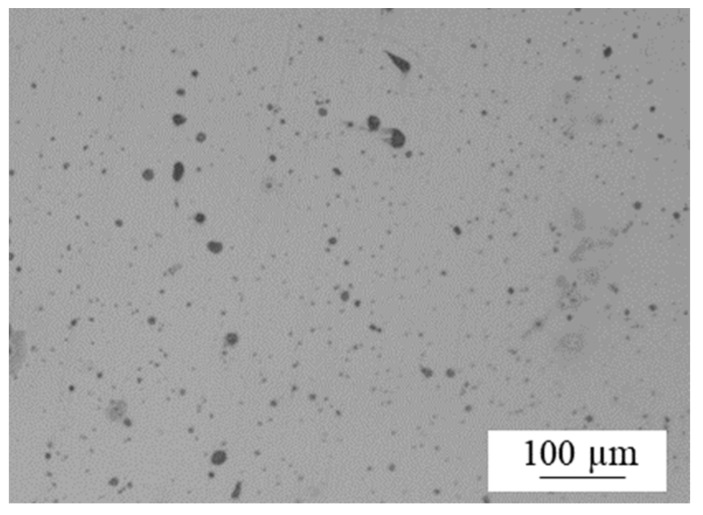
Micropores in the BMD manufactured parts.

In addition to the inter-raster voids, microporosity was also observed in the sintered parts, as represented in Figure 10. In the cross-sectional view, these pores generally had a circular shape with a maximum diameter of the order of ≤25 µm. These pores corresponded to residual porosity associated with the sintering process, which depends on the time–temperature combination in the sintering cycle.

**Figure 11:**
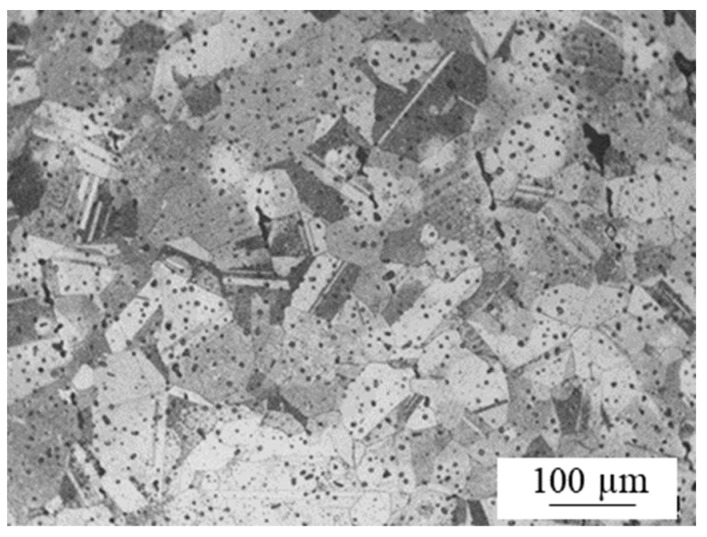
Microstructure of the sintered BMD parts.

As an example, Figure 11 shows the typical microstructure of 316L sintered parts. It can be observed that the microstructure is constituted by austenite grains with annealing twins. As the processing and raw materials were the same in all experiments, this microstructure was commonly observed in all cases.

### 3.3. Porosity

The porosity of the samples was analysed based on the Archimedes principle, and for each experiment, the density of nine small samples was determined. Porosity measured by this method included the micro and macro porosity observed in Section 3.2. The resultant mean and standard deviation values are presented in Table 2. The results show that the average porosity of the parts in experiments 1–4 was higher than 5% for all cases, and it was in the range of 5.58–6.66%. The effect of the build orientation can also be observed, as the vertical samples (experiments 2 and 4) had slightly higher porosity than the horizontal ones (experiments 1 and 3). Nonetheless, temperature did not seem to have a significant influence on the porosity value, since the horizontal and vertical parts manufactured at room temperature (experiments 1 and 2) and 50 °C (experiments 3 and 4) showed similar values.

On the other hand, parts manufactured in experiment 5 showed a porosity lower than 5%. Comparing the porosity values of horizontal parts (experiments 1, 3 and 5), it is clear that the infill pattern had a noticeable effect on porosity. This is the consequence of using the concentric infill pattern in experiments 1 and 3, as compared to experiment 5, which was performed with lines. The difference in the density of the parts can be related to the change of orientation between adjacent layers and its effect on the pore geometry as previously mentioned (Section 3.2). In experiments 1 and 3, the consecutive layers were printed with a little horizontal displacement, resulting in voids with triangular geometry. However, in the case of experiment 5, the consecutive layers were perpendicular to each other (±45°), thus producing elongated pores. The difference in pore geometry is thought to exert an influence that results in a lower porosity in experiment 5. Finally, the porosity values observed in this work agreed well with those reported by Desktop Metal [29], since it establishes a porosity of approximately 5% for 316L parts manufactured by BMD.

### 3.4. Vickers Microindentation Hardness

Three specimens were analysed for each of the experiments, and five microindentations were made in each of them. The average and standard deviation of the hardness tests for the five experiments are shown in Table 3. It can be seen that there were no significant differences in the average values of hardness. This may be due to the fact that the microstructure is austenitic in all experiments and that there are no great differences in microporosity.

The mean microindentation hardness value was around 120 HV, which was similar to the reported value by Desktop Metal [29]. However, Table 3 shows that this value was somewhat lower than the experimental values of 316L parts obtained by casting and PBF. The reported average hardness for the specimens fabricated by casting was 165 HV, whereas it increased to 230–280 HV for 316L-PBF parts. The lower values of BMD parts may be attributed to the presence of intragranular porosity and the collapsing of the pores in the material under load [30]. It must also be considered that due to the batch furnace configuration of the Studio System, an additional contribution to softening of the sintered material is the extremely low cooling rate. The hardness of the parts manufactured in this study may be improved by adjusting the temperature and time conditions of the sintering cycle in order to reduce microporosity [31].

**Table 3:** Mean and standard deviation values of the hardness for experiments 1–5.

Experiment/Condition	Mean Hardness (HV)	Standard Deviation	Reference
1 (X-room-concentric)	122	10	This study
2 (Z-room-concentric)	124	7	This study
3 ((X-50-concentric)	124	8	This study
4 (Z-50-concentric)	125	3	This study
5 (X-50-lines)	121	3	This study
316L-PBF	~230–280		[32,33,34,35]
316L-casting	~165		[33]

### 3.5. Tensile Test

**Figure 12:**
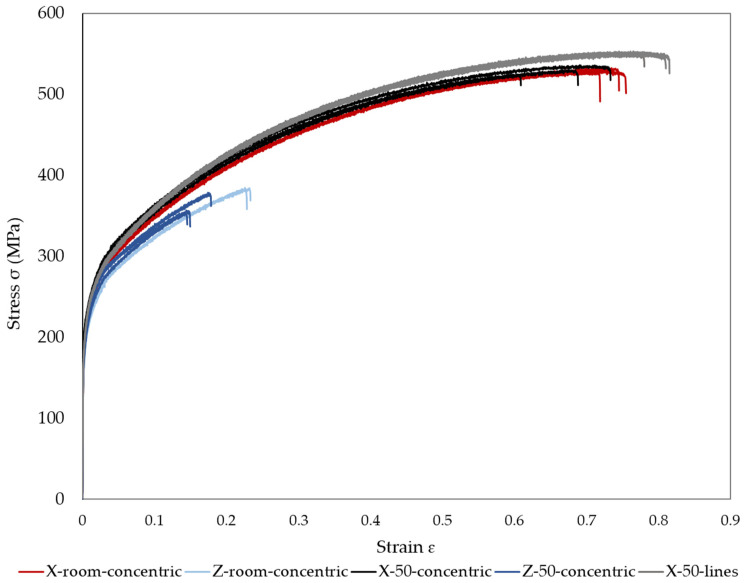
Stress–strain curves of machined coupons.

A total of fourteen tensile specimens were tested. The full stress–strain curves of the machined tensile coupons are plotted in Figure 12, while Table 4 shows the average yield strength ($\sigma_{0.2}$), ultimate tensile strength, elongation and area reduction for each of the conditions. It can be seen that the yield strength values of the five experiments are similar and are in the range of 182–199 MPa, although the values of horizontal parts are slightly higher than that of vertical ones. Furthermore, all the conditions show a higher yield strength than 165 MPa, the value provided by Desktop Metal [29].

Regarding the ultimate tensile strength, the results of the specimens with a concentric infill pattern (experiments 1–4) show that the build orientation of the parts had a noticeable effect on UTS. It can be appreciated that the ultimate tensile strength was considerably higher for horizontal parts (530 MPa for experiments 1 and 3) than for vertical ones (384 MPa for experiment 2 and 363 MPa for experiment 4). This difference may be attributed to the total porosity values and the distribution of the macropores. Regarding the porosity, Table 2 shows that horizontal parts showed a slightly lower porosity (5.58 and 5.76%) than the vertical ones (6.47 and 6.66%). It was reported in the literature that a higher porosity may result in a lower UTS [16]. With respect to the macropores, it can be observed in Figure 8 and Figure 9 that the distribution of the voids depends on the build orientation. The macropores with sharp edges may act as stress raisers and thus as crack initiation points. Furthermore, cracks may grow more easily in the case of vertical specimens because they extended along the interface between two adjacent layers, producing the final fracture. However, in the horizontal parts, although the air gaps may also act as crack initiation points, the cracks did not propagate so easily because they encountered the lines along the axial direction, which hindered their propagation. Therefore, the results showed that the build orientation had a significant effect on UTS.

With respect to the chamber temperature, it can be observed that this parameter does not have an appreciable effect on ultimate tensile strength. Although the general appearance of the samples showed that an increase in the temperature of the printing chamber produced an improvement in the adhesion between layers (Figure 6), its influence on tensile properties was not observed. This may be because the temperature change of ~25 °C was too small to notice a difference in tensile behaviour.

The infill pattern appears to have a slight effect on tensile strength because the difference between experiments 1 and 3 with respect to experiment 5 was 20 MPa, approximately. Table 2 shows that the porosity of experiment 5 was lower than in the other horizontal specimens (experiments 1 and 3). Furthermore, the micrographs of Figure 8 showed that the shapes of the voids in the first experiments were triangular, whereas in experiment 5, they were elongated as a consequence of the infill pattern. These differences in addition to the alignment of the voids may be responsible for the slight improvement in ultimate tensile strength, as reported in the literature [36].

The ultimate tensile strength values of horizontal BMD parts (experiments 1, 3 and 5) were slightly higher than the values for specimens fabricated by casting but significantly lower than the values for PBF parts (Table 4). This difference may be attributed to the presence of macropores and their distribution, which results in a decrease in mechanical properties. The size of these voids may be reduced by changing process parameters such as nozzle temperature, raster width and layer thickness [13,37]. It has been reported that increasing nozzle temperature may improve the adhesion between adjacent rasters and thus reduce the voids generated between them. With respect to macropore distribution, other infill patterns may result in different macropore distributions and/or macropore shapes and sizes, which may improve mechanical properties.

Therefore, future lines in the research of BMD process may be focused on the study of the influence of the process parameters aiming at obtaining an overall improvement of mechanical properties.

On the other hand, Table 4 and Figure 12 show that the stress–strain curves of horizontal parts (experiments 1, 3 and 5) presented typical curves of ductile materials with high elongations (74, 67 and 80%, respectively), whereas the stress–strain curves of vertical parts (experiments 2 and 3) exhibited lower elongations (15 and 23%, respectively), and thus, lower ductility.

**Table 4:** Elastic modulus, yield strength (σ_0.2_), ultimate tensile strength, elongation and area reduction mean values for each of the conditions.

Experiment/Condition	$\sigma_{0.2}(\mathrm{MPa})$	UTS (Mpa)	Elongation at Break (%)	Area Reduction (%)	Reference
1 (X-room-concentric)	189	530	74	41	This study
2 (Z-room-concentric)	182	384	23	12	This study
3 (X-50-concentric)	199	530	67	39	This study
4 (Z-50-concentric)	182	363	15	12	This study
5 (X-50-lines)	197	552	80	43	This study
316L-PBF		~640–750			[32,33,35]
316L-casting		~450–485			[33,38]

**Figure 13:**
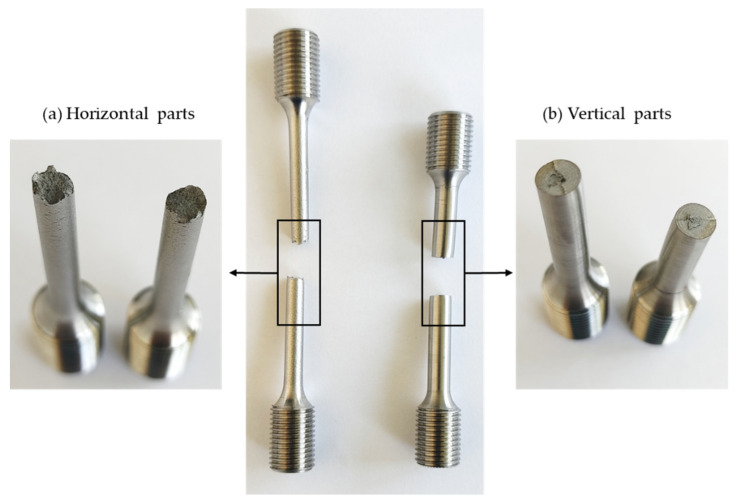
Fracture surface appearance: (**a**) horizontal parts; (**b**) vertical parts.

Figure 13 shows that the fracture surface morphology was very different for horizontal (experiments 1, 3 and 5) and vertical parts (experiments 2 and 4). The crack propagation in vertical specimens mainly occurred along the interface of two layers, producing a flat fracture surface. In contrast, in the horizontal parts, the crack had to cross different rasters, finally producing a rough fracture surface.

## 4. Conclusions

In this work, several 316L sintered specimens were successfully obtained by BMD using the Desktop Metal Studio System. Cylindrical test samples were printed in the horizontal and vertical directions with varying infill patterns and chamber temperatures.

The microstructural analysis revealed the presence of macropores, which were generated as a consequence of the air gaps formed between rasters during printing. The tensile test results showed that the shape and distribution of these macropores, which depended on build orientation and infill pattern, had a significant effect on the mechanical properties, especially ultimate tensile strength.

Although the horizontal (experiments 1 and 3) and vertical parts (experiments 2 and 4) with concentric infill showed voids with triangular shapes and sharp edges, the distribution of these defects varied with build orientation (Figure 8 and Figure 9). In addition, the porosity of horizontal samples was in the range of 5.58–5.76%, whereas it was slightly higher (6.47–6.66%) for vertical samples. As a consequence, the UTS of the horizontal specimens was approximately 160 MPa higher than in vertical parts.

Regarding the infill pattern, the effect of this parameter on the voids, and thus, mechanical properties, was also observed. As shown previously, the horizontal parts with concentric patterns showed triangular voids and a porosity higher than 5%, which resulted in a UTS of 530 MPa. However, samples with a lines infill pattern presented elongated macropores and a total porosity lower than 5%, characteristics that resulted in an improvement in UTS of 20 MPa, approximately.

Throughout the development of this study, it was possible to establish the relationship between some of the most important process parameters and properties of the parts manufactured by BMD technology. It was observed that the presence of voids and micropores had a significant effect on the mechanical properties of the metal parts printed using this technique. Therefore, future research lines for BMD should focus on reducing the presence of these defects by studying the influence of other process parameters such as nozzle temperature, raster width, sintering cycle or layer thickness.

## Figures and Tables

**Table 1:** Summary of the conditions used in the experiments.

Experiment	Build Orientation	Chamber Temperature	Infill Pattern
1	x axis	Room temperature	Concentric
2	z axis	Room temperature	Concentric
3	x axis	50 °C	Concentric
4	z axis	50 °C	Concentric
5	x axis	50 °C	Lines

**Table 2:** Mean and standard deviation values of the porosity for experiments 1–5.

Experiment	Mean Porosity (%)	Standard Deviation
1 (X-room-concentric)	5.58	0.48
2 (Z-room-concentric)	6.66	0.48
3 (X-50-concentric)	5.76	0.47
4 (Z-50-concentric)	6.47	0.28
5 (X-50-lines)	4.37	0.53

## Data Availability

The data presented in this study are available on request from the corresponding author.
